# High expression of *Snail1* is associated with EMAST and poor prognosis in CRC patients 

**Published:** 2019

**Authors:** Somayeh Mohammadpour, Amir Torshizi Esfahani, Raana Karimpour, Farbod Bakhshian, Seyed Abdolreza Mortazavi Tabatabaei, Asma Laleh, Ehsan Nazemalhosseini-Mojarad

**Affiliations:** 1 *Basic and Molecular Epidemiology of Gastrointestinal Disorders Research Center, Research Institute for Gastroenterology and Liver Diseases, Shahid Beheshti University of Medical Sciences, Tehran, Iran*; 2 *Department of Cellular and Molecular Biology, Faculty of Advanced Science and Technology, Tehran Medical Sciences, Islamic Azad University, Tehran, Iran*; 3 *Department of Food Sciences and Technology, Faculty of Pharmacy, Tehran Medical Sciences, Islamic Azad University, Tehran, Iran*; 4 *Proteomics Research Center, Faculty of Paramedical Sciences, Shahid Beheshti University of Medical Sciences, Tehran, Iran*; 5 *Gastroenterology and Liver Diseases Research Center, Research Institute for Gastroenterology and Liver Diseases, Shahid Beheshti University of Medical Sciences, Tehran, Iran *

**Keywords:** Snail1, Elevated microsatellite alteration at selected tetranucleotide repeats (EMAST), Survival, Colorectal cancer

## Abstract

**Aim:**

This study aimed to determine the link between Snail1 expression and CRC patients’ survival as well as its significant association with EMAST status.

**Background:**

*Snail1* is an evolutionary preserved zinc-finger transcription protein which contributes to Epithelial-to-mesenchymal transition (EMT). EMT initiates invasion and proliferation in many tumors. Elevated microsatellite alteration at selected tetranucleotide repeats (EMAST) is a marker of poor prognosis in patients with colorectal cancer (CRC). We hypothesized that *Snail1* overexpression is an important mediator of metastasis and decreased survival in CRCs that characteristically have EMAST phenotype.

**Methods:**

Quantitative real-time polymerase chain reactions were carried out to analyze the expression levels of Snail1 in both normal and tumor specimens from a total of 122 paraffin-embedded tissues (FFPE) of CRC sample with known EMAST status. The correlation between Snail1 expression and clinicopathological characteristics, survival, and EMAST status were examined.

**Results:**

Snail1 overexpression was detected in tumor tissues in 32% of all examined patients and its positive expression was related to metastasis (p=0.001) and EMAST+ phenotype (P=0.017). Further, positive Snail1 expression correlates with poor overall survival in CRC patients (P=0.01).

**Conclusion:**

Our findings suggest that *Snail1* overexpression is not only associated with EMAST but also with clinicopathological variables of poor prognosis. These results indicate that Snail1 expression levels may be useful for establishing novel therapeutic strategies and could help survival improvement in CRC patients.

## Introduction

 Epithelial – mesenchymal transition (EMT) participates in a biological process which acts as a paramount program within embryogenesis development, wound re-modeling (1) and is considered to be an indispensable element during alteration from benign tumor, where the tumor cells have not spread to nearby organs, into malignant tumor which offers the capacity of invasion of cancer cells (2, 3). Transition from epithelial state to mesenchymal characteristic through this biotic process is accompanied with cell junction’s degradation, loosening polarity and acquiring the ability of migratory properties required for intra-vastation of cells into vessels and extra-vasation into surrounding tissues which is called metastasis as alluded in recent findings (4, 5). In a cellular state, one of the pivotal steps in mechanism of distant metastasis involves EMT which is also the leading cause of cancer deaths such as in CRC (6). CRC is potentially a fatal disease, whereby EMT has been considered as a highly relevant issue that provide feature for carcinoma cells to a more aggressive phenotype and consequently results in CRC patient's death (7). Studies have illustrated that many transcription factors have demonstrated to be responsible for this well-defined EMT process consisting of Snail1, Slug, ZEB1, and Twist (8). Among these EMT-related transcription proteins, Snail1, a zinc finger transcription protein is fundamentally responsible for EMT(9, 10) and is the focal point of current study. EMT-driver transcription factor, Snail1, is an evolutionary preserved protein that contributes substantially to basal membrane dissolution by decreasing expression of E-cadherin away and enhancing mesenchymal genes (3, 11). In other words, the EMT program and the ability of invasion and metastasis of CRC cells are orchestrated by Snail1 family (11). The over-expression of *Snail1 *correlates with poor prognosis in CRC (12). Nevertheless, forthcoming evidence indicates that the notion of CRC patient’s survival being affected by EMT-inducers has remained a matter of debate (13).

Beyond Microsatellite instability (MSI) as a prognostic biomarker (14), elevated microsatellite alterations at selected tetra nucleotide repeats (EMAST), another form of MSI, is correlated with poorer survival in CRC patients (15). The molecular mechanisms of EMAST are unclear yet. Once MSH3, a member of DNA mismatch repair system, is translocated from the nucleus to cytosol, it causes more aggressive cell behavior, modifies the propensity to develop metastasis, and contributes to poor patient survival (16) as well as less sensitivity to 5‐fluorouracil (5-FU) based chemotherapy (17). Nonetheless, little is known about the biological value of EMAST in CRC (18).

The aim of this study is to evaluate the *Snail* expression as an EMT-related gene in CRC patient’s survival and its association with EMAST^+^ tumors. Almost certainly, to the best of our knowledge no data about the EMAST phenotype biology have been published. Thereafter, in this study we investigate the association between this phenotype serving as a biomarker and EMT-related genes expression. 

## Methods

A total of 122 patients with CRC who underwent surgery at Taleghani Hospital and Shohada Tajrish Hospital, Shahid Beheshti University of Medical Sciences between 2010 and 2017 and their EMAST status had been examined in Formalin-fixed paraffin-embedded (FFPE) tissues (19), were included in this study. The tumor and normal adjacent tissue were used for evaluation of *Snail* expression, and its association with survival and EMAST marker in CRC patients. Ethics approval of this study was obtained from the Medical Ethics Committee of Gastroenterology and Liver Disease Research Institute of Shahid Beheshti University of Medical Sciences.

**EMAST evaluation**

As mentioned above, the EMAST phenotype had been determined in a previous study in these patients (19). In brief, DNA extraction from normal adjacent FFPE tissues was performed by FFPE DNA extraction kit produced by QIAGEN GmbH (QIAGEN GmbH, Germany). Five tetra nucleotide markers including D9S242, MYCL1, D8S321, D20S82, and D20S85 were used for evaluation of EMAST. PCR optimization was performed by primers designed for each EMAST panel marker (19). We used QIAxcel capillary electrophoresis, High Resolution Cartridge, 25-500 nucleotide molecular markers, and 15-156 nucleotides align marker for detachment of segments generated by PCR and to compare microsatellite instability in tumor and normal samples of each patient (19). When at least two of five markers show a different pattern in tumor cells than normal, it is called EMAST positive (EMAST^+^). However, if only one or none of the markers in the tumor cells shows instability relative to adjacent normal cells, the sample is considered to be EMAST negative (EMAST).

**RNA isolation and gene expression analysis**

Total cellular RNA isolation from tumor and normal adjacent FFPE specimens was performed following the RNeasy® FFPE kit (QIAGEN, Germany), based on the manufacturer’s procedure. RNA proportion and quality analysis were determined with a Nano-Drop ND-1000 spectrophotometer (Thermo Scientific, USA). The extracted RNA reverse transcribed to complementary DNA (cDNA) using Prime Script-RT Master Mix (Takara Bio Inc., Otsu, Japan) and Random hexamer primers in accordance with manufacturer’s recommended protocol. Thereafter, synthesized cDNA samples were stored at -20℃. Detection of *Snail1 *was observed through Quantitative reverse transcriptase PCR (RT-qPCR) using the Light Cycler ABI 7500 Real-time PCR system and Maxima® SYBER Green/Rox with MicroAMP optical 96-well reaction (Applied Biosystems, USA) pellet. The qPCR amplification cycle was arranged in two stages as follows: at Holding stage, an initial denaturation at 95℃ for 30 sec, followed by 45 cycling stages consisting of denaturation at 95℃ for 5 sec, annealing at 60℃ for 34 sec and finally an extension at 72℃ for 30 sec. Notably, each sample was evaluated twice due to the fact that one of the pivotal issues in biological experiments is test repeating principle (20). The final reaction volume of 20μl contained 0.4 μl of each primer, 10 μl Maxima SYBER Green/Rox, and 4 μl of cDNA as PCR template. Additionally, the 2^−ΔΔCt ^(Threshold cycle) method was applied in order to normalize C_t _values to the reference gene, β-actin, and relative gene expression analysis. Note that primers for qPCR were designed at gene runner including *Snail1* (Forward primer: AAGGATCTCCAGGCTCGAAAG, Reverse primer: GCTTCGGATGTGCATCTTGA respectively), *β*-actin (Forward primer: CACCATTGGCAATGAGCGGTTC, Reverse primer: AGGTCTTTGCGGATGTCCACGT) and their efficiency was calculated using LinReg Software. The relative quantitation (RQ) values were used in statistical analysis. 

**Statistical analysis**

All computational analyses were accomplished using statistical software SPSS 16.0 and GraphPad prism 6.01. Further, all data were statistically analyzed using the Statistical Package for the Social Sciences, version 21.0. Differences in distributions between the variables were assessed via the Chi-Square test. Additionally, the comparison of gene expression in two different variables was performed using nonparametric independent samples T test and Mann Whitney test according to the resulting data. Kaplan-Meier curves for overall survival were created using GraphPad Prism software. Furthermore, a Long-Rank test was used to compare the survival curve groups. In all analyses, P values of less than 0.05 were considered statistically significant. 

## Results

**Figure 1:**
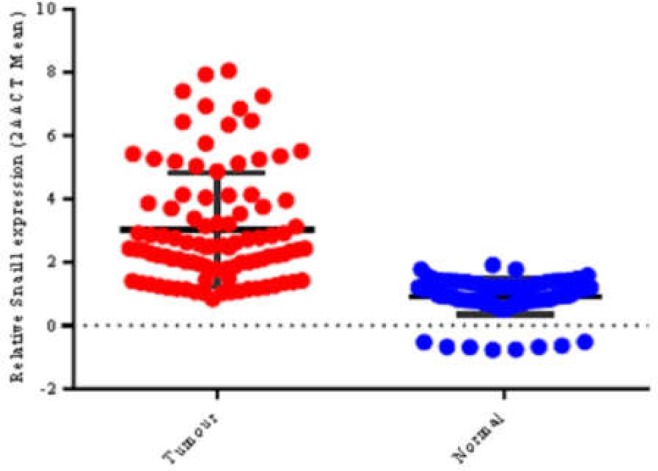
*Snail1* expression in CRC; *Snail1* expression was significantly higher in tumor specimens in comparison with normal adjacent tissue (p<0.001)

Of 122 specimens, 49 (40.2%) were EMAST^+^. *Snail1 *expression was significantly higher in tumor specimens compared to normal adjacent tissues (NATs) (P<0.001, Figure 1). The mean values of *Snail1* RQ in CRC tumors were 2.62±3.56 (median, 1.11), in 122 cases.

**Figure 2:**
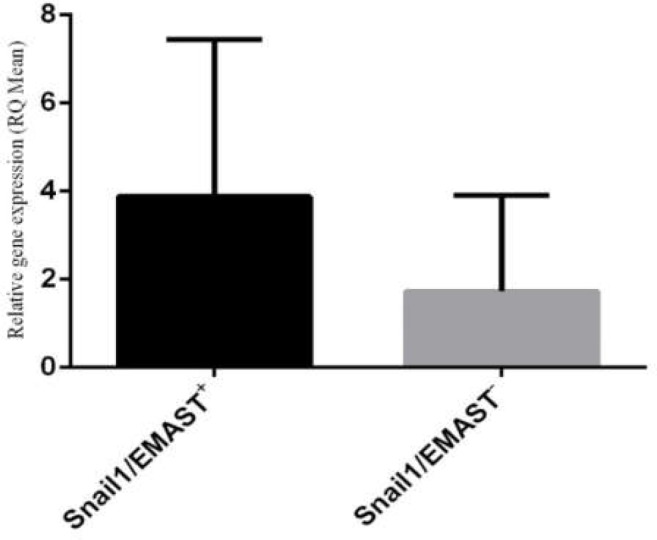
Correlation between relative mRNA quantification (RQ) of *Snail1* and EMAST status (*P*=0.017). RQ is expressed as fold-change using the 2^−ΔΔCt^ method in real-time PCR analysis

*Snail1 *overexpression was detected in tumor tissues in 32% of all examined patients (39/122). Table I summarizes the differences in the mean values of RQ of each marker according to the clinicopathological characteristics and EMAST status. The mean values of RQ of *Snail1 *were not significantly different in regard to stage and differentiation. Patients experiencing metastasis had significantly higher mean values of RQ for *Snail1 *rather than non-metastatic samples (p=0.001). Likewise, as depicted in Figure 2, The mean values of RQ for *Snail1 *observed to be higher in tumors characterized with EMAST^+^ phenotype (p=0.036). 

The mean values of RQ for *Snail1 *were significantly higher in patients with metastasis and EMAST phenotype. In addition, statistical investigations revealed that expression of *Snail1 *was significantly higher in tumors with EMAST ^+^phenotype (P=0.017).

**Figure 3:**
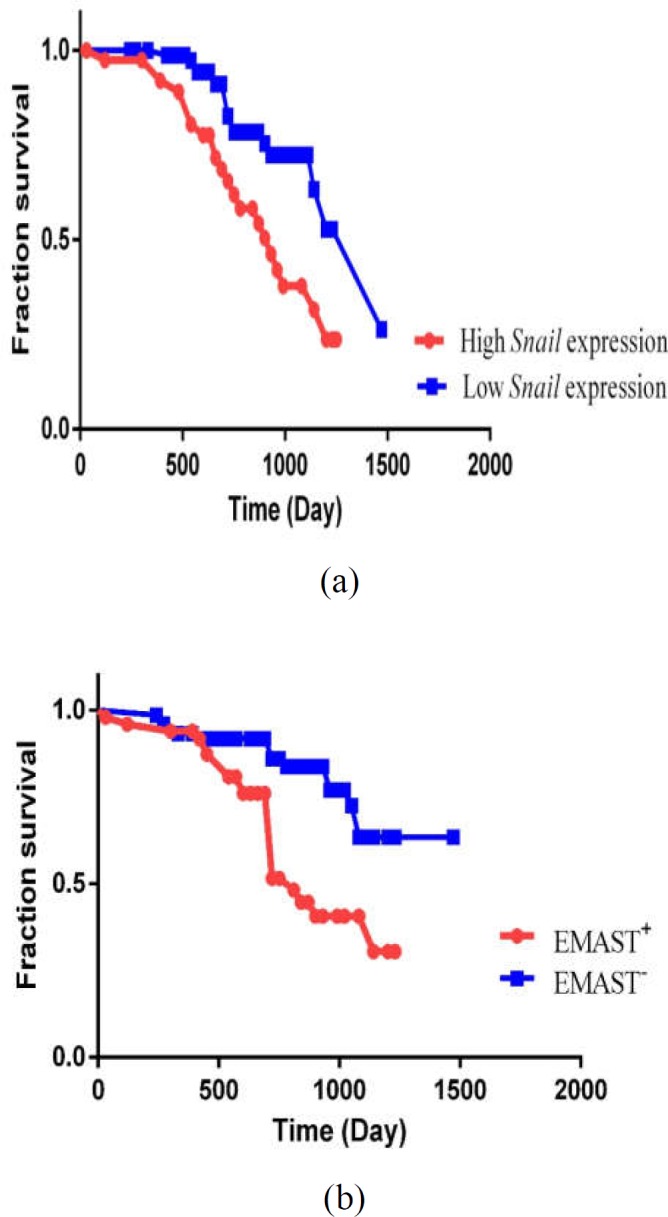
Kaplan–Meier curve of 122 CRC patients; (**A**) Survival analysis according to *Snail1* expression; Patients with higher expression of *Snail1* had poorer survival than those with lower expression of *Snail1 *(*P* = 0.01). (**B**) Survival analysis according to EMAST status; Kaplan-Meier univariate analysis demonstrated a significantly decreased survival probability in patients with EMAST+ phenotype (*P* =< 0.001)

Over the 50 months of follow-up (from range, 8-82 months), 39 patients (32%) had died. The median overall survival time by the entire patient cohort was 50 months, with a nominal 1-, 3- and 5-year survival of 96%, 88%, and 49%, respectively. As demonstrated in Figure 3, patients characterized with EMAST phenotype showed poorer overall survival (OS) (P= <0.001) where 48.97% of patients had died (24/49) while, only 20.54% of patients with EMAST^-^ had died (15/73). Further, the obtained data suggest that increased *Snail1 *expression was associated significantly with decreased OS (P=0.01), as compared to patients with low *Snail1 *expression.

## Discussion

EMAST is observed in 40-60% of CRCs. The clinical behavior of this instability at tetranucleotides tumors is distinctive, and the most intriguing and consistently described feature is metastasis and reduced survival (16, 21). The molecular basis for the prognostic disadvantage due to EMAST is not clearly established. Multiple studies have revealed that a key initial step in tumor metastasis is a molecular program called EMT which has been envisaged to be a chief event in cancer malignancy including CRC (22). We focused attention on a zinc-finger transcription factor expression, Snail1, the best transcriptional activator of EMT which can be considered as an essential element in tumor progression (12). Previous extensive studies demonstrated that 77% of colon cancer cells in stroma, stroma cells along with fibroblast phenotype in particular, had a higher expression of Snail1 (23). Snail1 expression in stroma highlighted that many cells have the capacity of escapism from the tumor initial site and notably a number of cells may penetrate into basal lamina in order to locate in the target organ (24). 

**Table 1:** Clinicopathological features of 122 patients according to *Snail1* expression

*Snail1 *Expression		Total	Characteristics
P-Value	Mean±SD	N (%)	
Tumor stage			
0.232	2.27±3.33 2.75±3.77	70(85.4%) 52(14.6%)	I-II III-IV
Differentiation			
0.517	2.32±3.1 2.72±3.81	40(48.8%) 82(51.2%)	Well Moderate/Poor
Metastasis			
0.001	3.92±4.02 1.6±2.88	52(14.6%) 70(85.4%)	Yes No
EMAST			
0.036	2.02±3.12 3.42±4.08	73(89.06%) 49(10.94%)	Negative Positive

Here, we demonstrate that increased instability at tetra nucleotides was associated with upregulation of Snail1 which is involved in downregulation of epithelial markers such as E-cadherin and activation of mesenchymal genes and consequently leads to carcinoma cell’s invasion (25). To the best of our knowledge, generally, this is the first study of Snail as an EMT-related gene expression in EMAST+ CRC tumors. In our analysis of 122 human colorectal cancer cases, Snail1 overexpression was associated not only with EMAST but also with clinicopathological variables of poor prognosis. The obtained results were broadly in line with previous studies which indicated that patients experiencing metastasis had a higher expression of Snail1 as compared to non-metastatic patients (26). In accordance with previous studies, there is a significant link among link expression in tumor’s with lymph node metastasis and overall survival in colorectal cancer. Interestingly, there is no evidence of any coordination of Snail1 expression with clinicopathological features including N-stage, grading, age or sex in CRC disease (27). Our findings are consistent with research showing that Snail1 overexpression is an exclusive feature that correlates significantly with poor survival in patients with CRC (28). In another study, Ziqian Li et al. suggested that Snail1 is not only a prognostic biomarker, but also has a predictive role. Further, fibroblasts characterized with Snail1 expression indicate CAF (chromatin-assembly factor) properties and through CCL1 (Chemokine C-C motif ligand-1) participation may lead to chemotherapy resistance of 5-fluorouracil/ paclitaxel in CRC. This casts a new light on the fact that inhibition of Snail1 expression fibroblasts in tumor can be a useful strategy in order to restrict chemotherapy resistance (29). As stated in a further research, Hector Peinado et al. unveiled that transforming growth factor beta (TGFβ) pathway is responsible for activation of promoter and induction of Snail1 expression in colon carcinoma cells. Further, the mitogen-activated protein kinase (MAPK) pathway seems to be directly involved in EMT network regulated by TGFβ1 in Madin-Darby Canine Kidney (MDCK) cells (30). Our study has suggested that EMT is impaired in CRC with EMAST+ compared to that with EMAST-. However, a signaling pathway according to EMAST status has not been identified. We hypothesized that variations in EMT signaling pathways might be one of the pivotal mechanisms that leads to prognostic differences in accordance with EMAST status. In the present study, Snail1 expression was correlated significantly with EMAST phenotype. Since one of the main features of EMAST CRCs is metastasis (16), these results provide evidence for the establishment of therapeutic strategies targeting EMT pathways according to EMAST status. CRC patients carrying EMAST characteristic have 5-FU therapeutic resistance (31). These CRCs are more susceptible to cancer metastasis. This study may be useful for developing new therapeutic strategies according to EMAST status and may be useful to improve survival outcome in CRC patients. However, investigating the morphological and functional differences of Snail1-induced EMT pathways in EMAST CRC in cell lines may help elucidate the molecular mechanisms of metastasis in CRC.
